# Reduced erythrocyte susceptibility and increased host clearance of young parasites slows *Plasmodium* growth in a murine model of severe malaria

**DOI:** 10.1038/srep09412

**Published:** 2015-05-06

**Authors:** David S. Khoury, Deborah Cromer, Shannon E. Best, Kylie R. James, Ismail Sebina, Ashraful Haque, Miles P. Davenport

**Affiliations:** 1 Complex Systems in Biology Group, Centre for Vascular Research, UNSW Australia, Kensington NSW 2052, Australia; 2 Malaria Immunology Laboratory, QIMR Berghofer Medical Research Institute, Herston, Brisbane QLD 4006, Australia

## Abstract

The best correlate of malaria severity in human *Plasmodium falciparum* (*Pf*) infection is the total parasite load. *Pf-*infected humans could control parasite loads by two mechanisms, either decreasing parasite multiplication, or increasing parasite clearance. However, few studies have directly measured these two mechanisms *in vivo*. Here, we have directly quantified host clearance of parasites during *Plasmodium* infection in mice. We transferred labelled red blood cells (RBCs) from *Plasmodium* infected donors into uninfected and infected recipients, and tracked the fate of donor parasites by frequent blood sampling. We then applied age-based mathematical models to characterise parasite clearance in the recipient mice. Our analyses revealed an increased clearance of parasites in infected animals, particularly parasites of a younger developmental stage. However, the major decrease in parasite multiplication in infected mice was not mediated by increased clearance alone, but was accompanied by a significant reduction in the susceptibility of RBCs to parasitisation.

It is estimated that in 2012 there were over 200 million cases of malaria globally, resulting in 627,000 deaths^1^. The majority of fatalities were thought to be caused by infection with *Plasmodium falciparum* (*Pf*)^1^. *Pf* infection can result in several severe syndromes, including acute respiratory distress, cerebral malaria and liver dysfunction^2^,^3^. Although the clinical symptoms of *Pf*-induced syndromes can differ markedly, they all display a strong association with high parasite load. Indeed, total parasite load is currently the best-known correlate of disease severity in *Pf*-malaria patients^3^,^4^. These data reinforce the central importance of reducing blood-stage *Pf* parasite loads to minimise disease severity in humans.

Experimental models of blood-stage malaria have been used extensively to identify host immunological and pathological mechanisms that may operate in humans^5^,^6^,^7^,^8^,^9^. One such model is the infection of inbred C57BL/6 mice with the rodent infective parasite, *Plasmodium berghei* ANKA (*Pb*A). Reports employing this animal model have provided much information on host factors contributing to disease severity and parasite load. However, assessments of host control of *PbA* infection have generally been restricted to measurements of parasitised RBCs in circulating blood (ie, parasitemia), or estimates of total parasite load^5^,^10^. Reductions in parasite level are generally attributed to increased host clearance of parasites, although this is not directly measured. Although previous *in vivo* studies attempted to measure clearance of circulating blood-stage parasites by using radio-labelled, infected RBCs^11^,^12^, these studies could not assess the importance of parasite clearance in controlling parasite multiplication rates (PMRs). Similarly, a recent study combining *in vivo* and mathematical modelling of parasite replication used only a very indirect approach to estimate the contribution of innate immunity to the control of parasite multiplication in blood-stage malaria^13^. Therefore, studies to directly determine the mechanisms of host control and the impact of parasite clearance on the overall dynamics of parasite multiplication in the blood-stream are required.

We have investigated the kinetics of *Pb*A parasite growth and clearance in C57BL/6 mice during the first week on infection. The rate of parasite multiplication in the blood-stream slows substantially over the early course of infection^14^, suggesting that early control of parasite growth in the blood-stream does occur in this model. However, the extent to which this is mediated by clearance of parasites from circulation remains almost completely unstudied. We hypothesized here that early control of parasite multiplication in the blood-stream in this model is mediated predominantly by active clearance of infected RBC from peripheral circulation. To test this *in vivo*, we developed novel methods for directly measuring host-mediated clearance of parasites from circulation. Using this approach, we demonstrated, firstly, that parasite multiplication rates dropped substantially during infection, and secondly, that parasite clearance increased during early *Pb*A infection, as the host acquired the capacity to clear parasites at earlier developmental stages. Nevertheless, increased parasite clearance alone does not fully account for reduced parasite multiplication. Instead, an accompanying phenomenon of altered RBC susceptibility, in which uninfected RBCs within an infected host became refractory to infection, acts to slow parasite multiplication during infection.

## Results

### Parasite multiplication rate is reduced in *Pb*A infection

**Figure 1:**
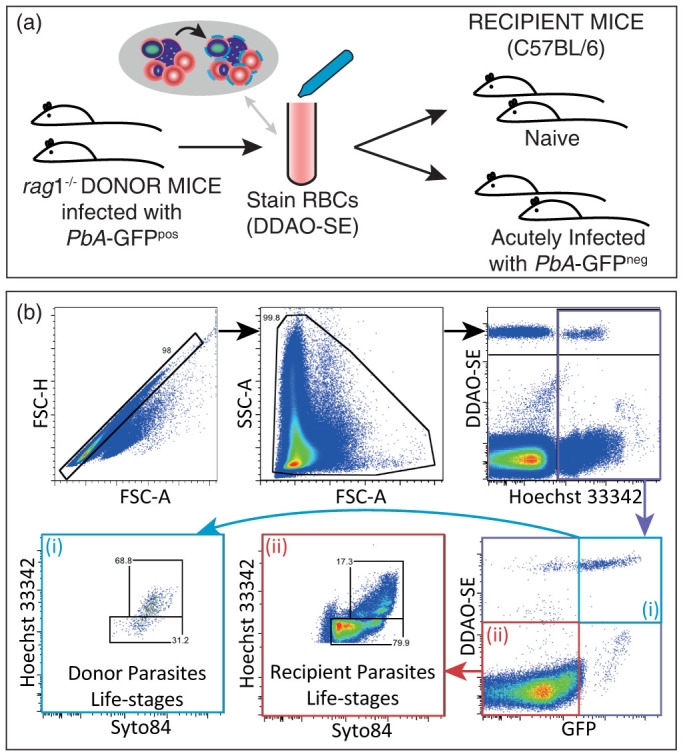
Experimental procedure and classificaiton of cells. (a) Donor mice (n = 2) were infected with transgenic *Pb*A-GFP^pos^ parasites 8 days prior to the beginning of the experiment. On day 0 of experiment, blood was taken from the donor mice by cardiac puncture, pooled together and fluroescently-labelled with the red blood cell (RBC) stain DDAO-SE. These labelled RBCs (donor RBCs) were then transferred into the two groups of mice, a group of uninfected (naïve) mice, and a group of mice that had been infected 5 days prior to infection with GFP^neg^ *Pb*A parasites. (b) Flow cytometry allowed RBCs to be distinguished from other cells based on size and structural complexity, using the measures of forward scatter (FSC) and side scatter (SSC). Doublets (two cells either adhering together or detected simulatenously) were excluded via FSC-Height (FSC-H) versus FSC-Area (FSC-A) plots. RBCs and parasites from the donor mice were distinguished from RBCs and parasites from the recipient mice by gating on GFP expression and DDAO-SE staining. Further, blood samples from mice were stained with Hoechst and Syto 84. This staining allowed late-stages parasites to be distinguished from early-stage parasites.

**Figure 2:**
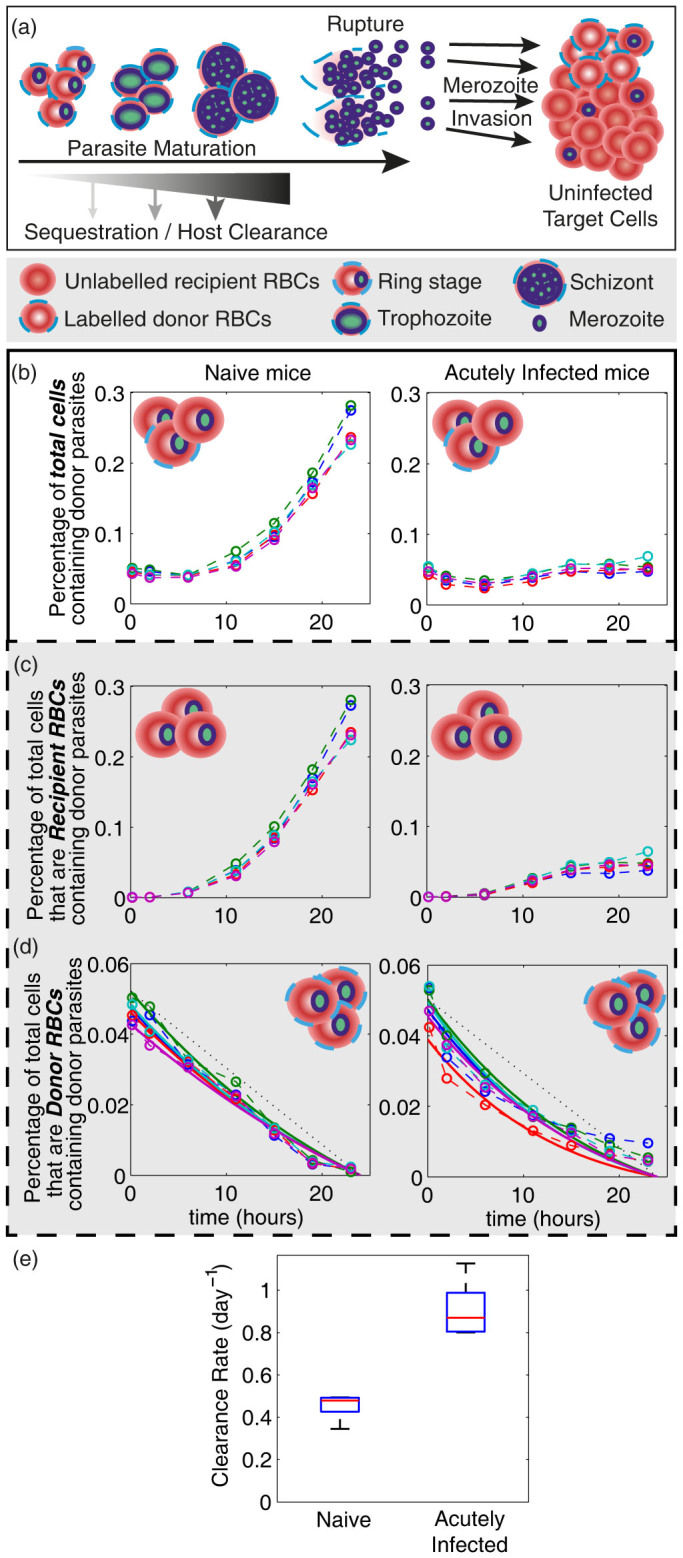
Donor parasites in recipient mice. (a) Once in the host, donor RBCs with GFP^pos^ parasites can be removed from the peripheral circulation of the host by parasite sequestration, host clearance of parasites, or parasite rupture (once the parasite reaches maturity). The donor (DDAO-SE^pos^) RBCs infected with donor (GFP^pos^) parasites that survive in the host until rupture release GFP^pos^ merozoites that are able to infect other susceptible target red blood cells. The uninfected target cells are predominately endogenous (recipient) RBCs, but there is a small portion (~1%) of uninfected donor RBCs. (b) Shows the percentage of total cells that are infected with donor parasites. This is the sum of: (c) the percentage of total cells that are recipient red blood cells and are infected with GFP^pos^ parasites; (d) and the percentage of total cells that are donor cells and infected with GFP^pos^ parasites. In (b) – (d) each color represents one mouse in each of the two groups. The circles and dashed lines indicate the observed % of total cells in each category. The solid lines in (d) are the results of fitting the model of parasite clearance (with age-independent clearance) to each of the mice, estimating the clearance rate for each mouse. (e) Shows a boxplot of the estimated clearance rates, of parasites for the naïve and acutely infected groups of mice obtained from fitting the model of parasite disappearance in (d). The red bars indicate the median estimate for each group, the blue boxes indicates the 25^th^ and 75^th^ percentiles, and the black whiskers indicate the range of clearance rate estimates.

Parasite multiplication rate (PMR) is an important determinant of total parasite burden in malaria infection. Our previous work suggested that PMR in the bloodstream drops over the course of acute *Pb*A infection in mice, although direct measures of this were not made^14^. Therefore, to measure PMR, naïve mice, and those infected 5 days previously with recipient (GFP^neg^) *Pb*A, were transfused with donor RBCs (fluorescently labelled with the dye DDAO-SE), and a proportion of which also contained donor (GFP^pos^) *Pb*A (Fig. 1a). The fold increase of circulating donor *Pb*A parasites over 24 hours was then monitored by frequent blood sampling and flow cytometric analysis (Fig. 1b), to assess rate of donor parasite invasion and growth in recipient (unlabelled) RBC, after rupturing out of fluorescent donor RBCs (Fig. 2a). The percentage of total cells that are infected with donor parasites (Fig. 2b) is equal to the sum of the percentage of total cells that are recipient cells infected with donor parasites (Fig. 2c) and the percentage of total cells that are donor cells infected with donor parasites (Fig. 2d). Over one parasite replication cycle (24 hours), donor parasites multiplied by 5.4 fold (range 4.7–6.2) in naïve mice (Fig. 2b). Surprisingly, however, donor parasites failed to multiply in the blood of 5-day infected (acutely infected) mice (fold change 1.0, range 0.9–1.2) (Fig. 2b). These data confirm that the parasite multiplication rate of *Pb*A drops substantially by the fifth day of *Pb*A infection in mice.

### Parasite clearance rates increase during *Pb*A infection

By 5 days post-infection mice exhibit a moderately enlarged spleen, and higher systemic levels of the macrophage-activating cytokines, IFNγ and TNF^15^. Therefore, we hypothesized that the reduced PMR observed 5 days post-infection was due to increased parasite clearance. To test this we tracked the loss of infected donor RBCs (*i.e.* fluorescently labelled RBC containing donor (GFP^pos^) *Pb*A (Fig. 2a)) from the blood of naïve and acutely infected recipient mice (Fig. 2d).

We observed a slightly increased disappearance rate of donor parasites from the circulation of the acutely infected mice, compared with the naïve mice (Fig. 2d). However, since *Pb*A has a known, 24-hour life-cycle, we first needed to account for donor parasite rupture in our decay curves, and then estimate the rate of disappearance from processes other than simply maturation. We fitted a mathematical model of the parasite life-cycle, which included parasite maturation and rupture, as well as the rate of disappearance of parasites due to active host clearance and/or sequestration into peripheral tissue microvasculature (Fig. 2d). These fits confirmed our hypothesis that the loss of donor parasites from circulation was faster in infected mice (clearance rate 0.87 day^−1^, range 0.80–1.1) compared to naïve controls (clearance rate 0.48 day^−1^, range 0.35–0.49) (Fig. 2e).

The faster loss of donor RBC containing donor parasites from the circulation of infected mice compared to naïve controls could theoretically have been due to increased clearance and/or increased sequestration of parasites. However, parasite sequestration is expected to decrease parasite clearance (by avoiding the need for passage through the spleen), and thereby increase parasite amplification over each infection cycle^7^,^14^,^16^, while clearance should reduce parasite numbers. As mentioned above, donor (GFP^pos^) parasite multiplication rates were substantially reduced in acutely infected mice, which suggests that donor parasites were lost due to increased parasite clearance, and not simply because of enhanced parasite sequestration.

### Infected animals clear younger parasites more effectively

Thus far, we had assumed in our mathematical models that parasite clearance uniformly affected parasites of all ages. However, since mature stage parasites may be cleared more effectively than early parasite stages *in vivo*^17^, we next modified our model such that clearance affected parasites only above some age, *x_c_*. We then fitted our model to the decay data for each group of mice, allowing parasites to become susceptible to clearance at a variety of different ages. We found that clearance of parasites aged ≥18 hours most closely reflected our *in vivo* data for naive mice, while, surprisingly, an age of ≥0.5 hours provided the best fit for data from acutely infected mice. Thus, our mathematical model predicted that early-stage parasites were cleared faster by acutely infected mice than naïve mice.

**Figure 3:**
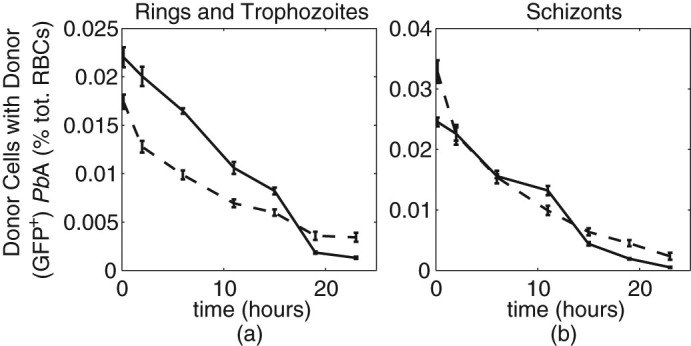
Faster clearance of immature parasites in the acutely infected mice than in naïve mice. The percentage of total cells that are donor red blood cells (RBCs) infected by GFP^pos^ (a) rings or trophozoites, and (b) schizonts are shown for naïve mice (solid line), and the acutely infected mice (dashed line). Each group contain n = 5 mice, and the mean for each group is plotted (error bars indicate standard error of the mean).

To test this prediction, we directly compared the rate of disappearance of different parasite life-stages in naïve and infected animals. Using fluorescent nucleic acid detection dyes, Hoechst 33342 to stain DNA, and a cell permeant RNA/DNA dye, Syto®84, in conjunction with flow cytometry, we classified parasites into developmental stages in a high-throughput, quantitative, manner^18^,^19^,^20^ (Fig. 1b). Applying this to donor RBCs containing donor parasites, we noted that mature stage parasites were readily observed, and decayed similarly in both groups of mice (Fig. 3a). Notably, however, younger life stages (rings and trophozoites) disappeared faster from the circulation of acutely infected mice compared to naïve controls (Fig. 3b). Thus our *in vivo* data were consistent with predictions from our mathematical modelling, and suggest that naïve mice primarily clear late-stage parasites, while infected mice can target younger parasites for clearance.

### Improved host clearance does not fully account for the substantial slowing of *Pb*A parasite multiplication in circulation

**Figure 4:**
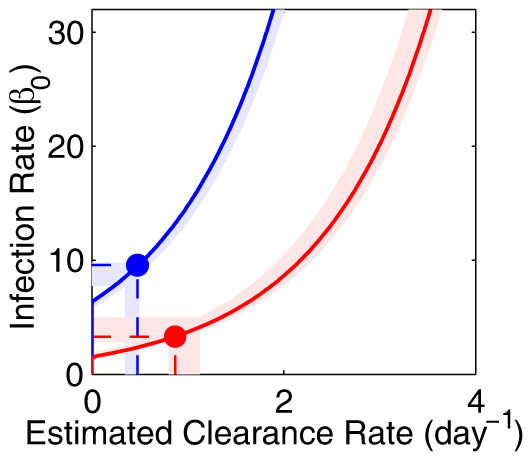
Estimating the parasite multiplication rate in naive and acutely infected mice. Fitting the data on the growth of the GFP^pos^ *Pb*A parasite population with the model of parasite growth with age-independent clearance yields these estimates of parasite clearance, which depend on the multiplication rate, *β*_0_ (solid lines are the median for each group, shaded regions indicate the range of estimates within each group). For each value of *β*_0_ clearance rates for the acutely infected mice are higher than those for the naïve mice. When fitting the disappearance curves of infected donor cells from the same hosts we estimated a clearance rate for each group of mice (dashed lines are the medians for each group). The points of intersection of the solid and dashed lines for each group of mice provide an estimate of the parasite multiplication rate *β*_0_ for each group of mice. We observe that a lower parasite multiplication rate is estimated in the acutely infected mice than in the naïve mice.

Since parasite clearance increased over the course of infection, we next determined whether this was sufficient to account for the five-fold reduction in PMR in acutely infected mice compared to naïve controls (Fig. 2b). PMR describes the net growth of parasites in the bloodstream, which is the rate of infection of RBCs minus the rate of parasite clearance. After taking into account possible confounding effects of donor cell handling and labelling on parasite viability (Supplementary Information 1.1), and using our estimates of parasite clearance rates (Fig. 2e), we found it impossible to fit the *in vivo* PMR data to our model using a single value for the infection rate (*β*_0_) across both groups of mice. This was evident when we fixed the infection rate (*β*_0_) to be a series of values between 0 and 32, and, fitted our model of parasite growth to the experimental parasite growth data estimating the corresponding clearance rate necessary to fit the growth data for each infection rate (Fig. 4). Using the clearance rates we estimated earlier (Fig. 2e) we observed that in order to fit the growth data different infection rates were required for each group (Fig. 4, intersection of solid and dashed lines), estimated at 9.5 (range 7.7–9.8) for naïve mice, and 3.3 (range 2.8–5.0) for acutely infected mice. This same approach was repeated using the model in which the age at which parasites were cleared was variable. Using the estimates for parasite clearance age and clearance rates for each group, we again observed a higher RBC infection rate in naïve mice compared with acutely infected mice (Supplementary Fig. 3). Thus, our modelling suggested that the differences in infection kinetics in naïve and acutely infected mice could not be accounted for simply by increased parasite clearance, but that reduced parasite infectivity would also be required.

### Relative contribution of parasite clearance and reduced infection rates in controlling parasite multiplication

Since our modelling suggests that both increased parasite clearance and reduced rate of infection of RBCs were required to fit the experimental data, we next assessed their relative contribution to controlling parasite multiplication in acutely infected mice. The PMR over one parasite multiplication cycle is given by
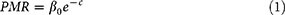
where *β*_0_ is the infection rate of RBCs and *c* is the clearance rate of parasites. Using this relationship we can see that comparing the PMR in naïve and acutely infected mice gives,
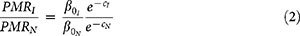
and so,
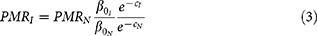
Therefore, the drop in PMR from 5.4 (range 4.7–6.2) in naïve mice to 1.0 (range 0.9–1.3) in acutely infected mice, is given by the drop in the rate of infection of RBCs (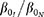), multiplied by the exponential of the increase in the clearance rate of parasites (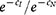). Using our estimates of parasite clearance (Fig. 2e) and the rate of infection of RBCs (Fig. 4) we noted that the increase in parasite clearance accounted for a ~32% reduction in PMR between acutely infected mice and naïve mice, while the drop in the infection rate accounted for a further 65% drop in PMR. Hence, we estimated that the reduced rate of infection of RBCs in acutely infected mice was twice as important as the increased parasite clearance at lowering PMR. The same calculations, when performed using estimates from the age-dependent model of parasite clearance, suggested that nearly all the reduction in PMR in the acutely infected mice was due to the reduced rate of infection of RBCs, with relatively little contribution made by enhanced parasite clearance mechanisms. Together, our analyses suggested that during *Pb*A infection, a reduction in the rate of infection of RBCs was substantially more important for controlling PMR than the observed increase in parasite clearance rates.

### Target cell (RBC) susceptibility is reduced in infected mice

**Figure 5:**
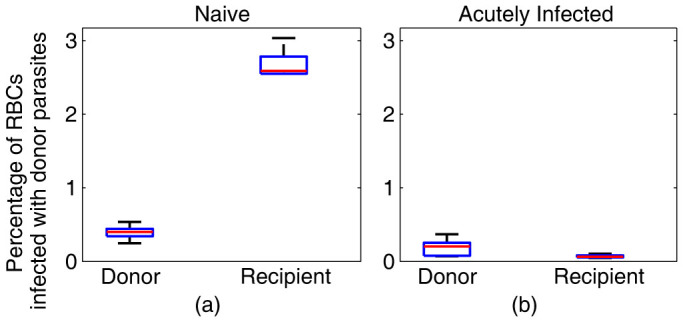
The susceptibility of red blood cells to infection is reduced in acutely infected mice. Uninfected recipient and donor cells can both be infected by merozoites released from a rupturing donor parasite. The percentage of donor (fluorescently labelled RBCs) and recipient (endogenous) RBCs that are infected with GFP^pos^ *Pb*A parasites 48 hours after donor red blood cells were transfused in (a) naïve and (b) acutely infected mice. The red line indicates the median for each group of mice (n = 5), the blue box provides the 25^th^ and 75^th^ percentiles, and the bars indicate the range of values. We see a very high preference for infection in endogenous (recipient) RBCs in naïve mice over the donor RBCs, and low infection of endogenous and donor RBCs alike, in the acutely infected mice. We note that there is a lower percentage of donor RBCs infected with GFP^pos^ *Pb*A parasites in the acutely infected mice compared to the naïve mice. This is due to the increased parasite clearance in acutely infected mice. Further, the percentage of endogenous RBCs infected in acutely infected mice is lower than the donor RBCs, suggesting that there is some reduced susceptibility of the endogenous cells to invasion.

Given our modelling suggested a pivotal role for reduced parasite infectivity in controlling parasite multiplication, we next investigated whether this might be due to reduced RBC susceptibility in the *Pb*A infected animals. Given the fixed 24-hour life cycle of *Pb*A parasites, we expect all donor parasites will have completed one cycle approximately 24 hours after transfusion. Therefore, one approach to investigate infectivity would simply be to compare the parasitemia of donor parasites in recipient cells after one cycle of infection. However, a reduced parasitemia might occur either due to infected RBC clearance, merozoite clearance, or reduced RBC susceptibility. To directly compare RBC infection rates in naïve and acutely infected mice, we utilised a fortuitous feature of our system – the presence of uninfected donor cells. These uninfected donor cells provide a reference population of RBC that is the same in both naïve and infected animals. Factors such as increased merozoite clearance in infected animals would affect the parasitemia in both donor and recipient RBC equally. However, if we compare the donor parasite levels in donor cells and recipient cells in the same animal, we can directly compare the susceptibility of these two cell types, exposed to the same number of merozoites. In naïve mice, we found that endogenous (recipient) RBCs were significantly more susceptible to infection than the donor RBC, since the parasitemia in recipient RBC was >6-fold higher than in donor RBC by 48 hours after transfusion (2.7% versus 0.4%, *P = * 0.008) (Fig. 5a). In stark contrast, in acutely infected mice, endogenous (recipient) RBCs appeared much less susceptible to infection than donor RBC, with recipient RBC showing only one third the parasitemia of donor RBCs (0.07% versus 0.19%, *P = * 0.06) (Fig. 5b). Although the parasitemia of donor cells differed between naïve and infected animals (due to the altered infection dynamics), we can still use these cells as a reference. This analysis indicates that endogenous RBCs in acutely infected mice were ~18 times less susceptible to infection than RBC in naïve mice. Taken together, our data suggest that exposure to the first 5 days of *Pb*A infection renders the uninfected RBC largely refractory to new infection by *Pb*A parasite, and furthermore, that this plays a major role in reducing parasite replication.

## Discussion

Uncontrolled multiplication of *Plasmodium* in host RBCs leads to high parasite loads, and contributes to the morbidity and mortality of malaria. Determining the mechanisms of effective parasite control *in vivo* may aid the development of novel strategies to reduce the incidence and severity of malaria. Here, we have presented *in vivo* evidence that a mammalian host exhibits a degree of control over early parasite multiplication via at least two concurrent mechanisms – increased parasite clearance and reduced RBC susceptibility.

Murine models provide a system in which the host's response to infection can be manipulated, and subsequent effects on parasite infection dynamics and disease can be explored. Numerous studies of early immune-mediated control of parasites have been conducted in rodent models of blood-stage malaria^5^,^6^,^7^,^8^,^9^,^10^,^21^,^22^,^23^,^24^. These studies identified several immune organs, factors or processes, including type I IFN signalling^21^, CD4^+^ T cells^5^, IFN-γ^10^ and the spleen^5^, which can affect the total parasite load in a host. However, the mechanisms by which host-immune factors affect parasite multiplication are rarely investigated. For example, although parasitemia and total parasite load are often measured, these net parasite loads may be controlled by two non-mutually exclusive mechanisms, reducing rates of infection of RBCs or increasing parasite clearance. Simply measuring net parasite burdens over time does not differentiate between these possibilities.

Dissecting the relative roles of increased clearance and impaired infection rates in controlling parasite loads requires that at least one of these factors be measured directly. Here, we developed novel *in* *vivo* and mathematical tools to study a) the rate of disappearance of parasites from circulating blood, and b) the rate of infection of RBCs, using a mouse model of blood-stage malaria. We demonstrated that a major decrease in the rate of infection of RBCs contributed substantially to the slowing of parasite multiplication late in infection, and was likely the result of less susceptible RBCs *in vivo*. In contrast, although parasite clearance certainly increased over the course of infection, this process contributed relatively little to parasite control.

Previous studies have attempted to measure the clearance rate of malaria parasites *in vivo*. Two studies used radio-labelled, infected erythrocytes in *Plasmodium berghei*^11^ and *Plasmodium chabaudi*^12^ infections. Interestingly, the former study, though finding parasite clearance was increased in immune versus non-immune mice, found this increase was relatively small, as was the case in our study. Further, the latter of these studies found that splenic uptake of parasitised erythrocytes did not greatly increase between the early-stage and the resolution stage of infection. This study in *P. chabaudi* infection suggested that splenic clearance was not the major mechanism responsible for resolving infection, and is consistent with the relatively small effect of clearance on *Pb*A infection observed in this paper. Both these studies and our study suggest that host clearance is relatively mild in controlling parasite multiplication, and that other changes during infection play a significant role in controlling parasite multiplication.

More recently, Metcalf *et al.* combined experimental analysis and mathematical modelling to examine how innate immunity might control parasite multiplication in malaria infections, by measuring infected and uninfected RBC concentrations^13^. However, they did not directly measure clearance and infection rates, and instead attempted to quantify changes in infection rates by assuming that RBC concentration was a direct indicator of the efficacy of invasion by parasites. Importantly, our work argues against this assumption, since we have demonstrated that major changes in RBC susceptibility can occur independent of RBC number. Studies have also attempted to estimate parasite clearance in human infection, examining the rates of the clearance of parasite life-stages under drug-treatment, including studies of the impact of asplenia on parasite removal^25^,^26^,^27^. However, the methods in these studies only allow clearance to be measured when an infection is in the process of being resolved. *Ex vivo* studies have also been used to determine rates of retention of infected RBCs in an isolated human spleen^17^, showing higher rates of retention of mature stage parasites compared with immature stages. Our study provides direct in vivo measurements of parasite clearance, even when an infection is not being resolved, and our methods are able to distinguish clearance from other mechanisms acting to remove parasites from circulation. As such we were able to directly characterise the important mechanisms in the control of infection.

A potential limitation in this study was the use of donor cells from infected *rag*1 deficient mice, which were also handled during labelling and therefore not identical to recipient cells. This may have affected the susceptibility of donor cells to invasion. For this reason we used the susceptibility of donor cells, which were identical in naïve and infected animals, as a standardized population to which we could compare the susceptibility of endogenous cells (Fig. 5). Similarly, handling may have led to a reduced viability of late-stage donor parasites following transfusion (Supplementary Information 1.1), although our modelling was able to take this into account.

The mechanisms of reduced susceptibility of RBCs in infected animals are unclear. Infection may damage host RBCs or reduce expression of surface proteins required for merozoite invasion^28^. Alternatively, an important possibility is that RBCs may exhibit a distribution in their natural susceptibility to infection. The most susceptible cells may be targeted and destroyed early in infection, leaving only relatively poor cells later. This explanation seems likely given the known preference of *Pb*A infections for reticulocytes^29^, and our previous observations that reticulocytes are greatly depleted by 4-days into *Pb*A infection in mice^14^.

The findings from our study are specific to early acute infection and hence innate host control mechanisms. However, the methods used in this work to dissect parasite clearance from other host control mechanisms are likely to be useful in studying adaptive immune responses. Experimental models of malaria infection where immunity can be generated^30^,^31^ will be examined in the future to reveal the mechanisms controlling parasite loads in these hosts. Understanding the mechanisms of host control of parasite multiplication is fundamental to the development of new approaches to reduce parasite burdens and disease severity in malaria. Our work has revealed that although host-mediated clearance of parasites increases during infection, it is not the only factor controlling parasite burden in a mouse model of severe and fatal malaria. Factors that modulate the rate of invasion of red blood cells by parasites play a major role in host control of infection.

## Methods

### Adoptive transfer and monitoring of “Donor” RBC populations

#### Mice and ethics

Female C57BL/6 mice aged 6–12 weeks were purchased from the Australian Resource Centre (Canning Vale, Perth, WA, Australia) and maintained under conventional conditions. C57BL/6J *rag1^-/-^* mice were bred and maintained at QIMR Berghofer Medical Research Institute. This study was carried out in strict accordance with guidelines from The National Health and Medical Research Council of Australia, as detailed in the document *Australian Code of Practice for the Care and Use of Animals for Scientific Purposes*, 7th edition, 2004. All animal procedures and protocols were approved (A02-633M) and monitored by the QIMR Berghofer Medical Research Institute Animal Ethics Committee.

#### Parasites and infections

*Plasmodium berghei* ANKA (*Pb*A) strains were used in all experiments after a single *in vivo* passage in wild-type C57BL/6 mice. A transgenic *Pb*A (231c1l) line expressing luciferase (*Pb*A*-*luc) under the control of the EF1-α promoter was used to infect wild-type (recipient) animals. Transgenic *Pb*A-GFP strains were maintained as previously reported and used to infect donor (*rag*1*^-/-^*) animals^22^. All mice were infected with 10^5^ pRBCs intravenously (*i.v.*). via the lateral tail vein.

#### Adoptive transfer of donor RBC

Donor *Pb*A-GFP-infected *rag*1^-/-^ mice were euthanized, and cardiac punctures performed to collect blood. Heparinised blood was washed twice in Ca^2+^/Mg^2+^-free phosphate buffered saline (PBS-A), and stained in CellTrace™ Far Red DDAO-SE (Life Technologies) according to manufacturer's instructions. Briefly, 50 μg CellTrace™ was dissolved for ten minutes in 25 ul dimethyl sulphoxide (DMSO). This was added to 5 ml of resuspended blood in PBS-A. Blood was stained in the dark, at room temperature with constant rolling for 15 minutes, and then washed twice in 10× volumes of PBS-A. Successful labelling of RBC was confirmed by flow cytometry using an LSRII Fortessa analyzer (BD Biosciences) and FlowJo software (Treestar, CA, USA). CellTrace™-labelled blood was resuspended in 2 ml volumes per donor mouse, and injected in 200 μl volumes via *i.v.* injection using a 26 G needle.

#### Flow cytometric analysis of blood

Forward scatter (FSC) and side scatter (SSC) were used to distinguish RBCs from other cell types. Plotting FSC-Area (FSC-A) and FSC-Height (FSC-H) allowed the exclusion of doublets (events recorded by the flow-cytometer that are the result of two cells being detected simultaneously) (Fig. 1b). A flow cytometric method, adapted from various research groups^18^,^19^,^20^, was employed to simultaneously detect adoptively transferred (CellTrace™-labelled) RBC, to distinguish GFP^pos^ (donor) from GFP^neg^ (recipient) parasites, and to ascertain parasite life cycle stages. Briefly, a single drop of blood from a tail bleed was diluted and mixed in 200 μl of RPMI medium containing 5 U/ml heparin sulfate. Diluted blood was simultaneously stained for 30 min in the dark at room temperature with the cell-permeant RNA/DNA stain, Syto84 (5 μM; Life Technologies) and with DNA stain, Hoechst 33342 (10 μg/ml; Sigma). Staining was quenched with 10 volumes of RPMI medium, and samples immediately analyzed by flow cytometry using an LSRII Fortessa analyzer (BD Biosciences) and FlowJo software (Treestar, CA, USA). Adoptively transferred donor RBCs were readily distinguished from endogenous RBC by CellTrace™-labelling (Fig. 1b). Infected RBCs were detected as being Hoechst 33342^+^ and Syto84^+^. Parasite stages were determined as shown in Fig. 1b.

### Mathematical Models and Fitting

#### Model of parasite disappearance

To interpret results from RBC adoptive transfer experiments we constructed a series of mathematical models, based on our previous study^14^, which were then fitted to the experimental data. We first constructed a model for the disappearance from peripheral circulation of fluorescently-labelled (donor) RBC, containing GFP^pos^ parasites (donor parasites). This model is given in equations (4) and (5). 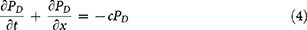

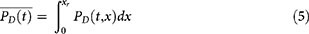
*P_D_(t,x)* is the density of donor RBCs containing donor parasites, of age *x*, at a given time *t* after transfusion (in days) and *c* is the rate of parasite disappearance due to host-parasite interactions. The total number of parasites at time *t* is given by, 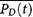. *x_r_* is the age at which parasites rupture in days (*x_r_* = 1 day for *Pb*A parasites^32^). Thus, this model considers the two mechanisms causing donor parasites to disappear from the peripheral circulation: 1) host cell interactions leading to clearance or sequestration; and 2) RBC rupture as parasites reach maturity (Fig. 2a). Since we are modelling events in the first 24 hours after transfusing parasites, *t* ≤ 1 day in all our modelling. This model is similar to our previous age-structured model of parasite populations^14^. We assumed that a uniform distribution of parasite ages was initially transfused into the recipient mice (consistent with the spread of life-stages observed in the donor parasite population from *rag*1^-/-^ mice). That is, 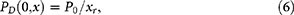
where *P*_0_ is the initial number of RBCs that are transfused into the recipient mice. We also assumed that the invasion of uninfected, donor RBCs by donor (GFP^pos^) parasites once inside recipient animals was negligible. This assumption was based on the fact that donor RBCs constitute only a small proportion of total RBCs. Hence, when a donor parasite reaches maturity within a donor RBC and ruptures, the resulting merozoites have many more recipient RBCs to infect compared with donor RBCs. This assumption provided a boundary condition for our model,
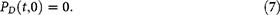
Solving the partial differential equation in equations (4) and (5) with boundary conditions in equations (6) and (7) yields the solution, 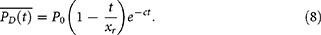
The factor 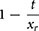 describes total parasite numbers decreasing due to parasite rupture, and the factor *e^−ct^* describes parasite numbers decreasing due to host-parasite interactions.

#### Model of parasite growth

The ability to identify donor parasites based on GFP expression, allowed us to measure a second crucial parameter, the rate of infection of RBCs with donor parasites within the recipient mice, after the parasites ruptured from fluorescently labelled donor RBCs. To analyse *in vivo* data related to the rate of infection of RBCs with donor parasites, we applied a similar model to that used above. However, this model also allows for new infection of RBCs by parasite invasion. To do this we modified the boundary condition (from equation (7)) in this model. Once a parasite is fully mature in a RBC, the cell ruptures releasing merozoites into the blood, which are able to infect other RBCs (Fig. 2a). Since merozoites have very short half-lives relative to the duration of our experiments^32^,^33^,^34^,^35^, we assumed the time between rupture and new infection of RBCs to be negligible (as in our previous model^14^). We assumed that the number of newly infected recipient RBCs is proportional to the number of rupturing parasites (of age *x_r_* day), which provided the boundary condition,
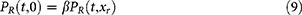
where *β* is the rate of infection of RBCs (the average number of RBCs that become infected from every rupturing parasite).

We also allowed for a decrease in the viability of transfused donor parasites with their age at the time of their initial transfusion, such that parasites that were older at the time of transfusion had a reduced viability. This is consistent with an observation in the experimental data (Supplementary Information 1.1, Supplementary Fig. 1 & 2). We do this by taking *β* to be a function of time *β = β(t)*, with
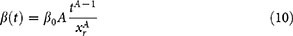
where *β*_0_ is the per cycle replication rate of the total parasite population, *x_r_* is the age of parasite rupture (*x_r_* = 1 in this paper), and *A* parameterises the change in viability of the transfused parasites changes with the age of parasites at transfusion (*A* = 2.6, see Supplementary Information 1.1). The estimated viability of parasites of different ages at transfusion is shown in Supplementary Fig. 2b.

#### Model of clearance of parasites of different ages

So far we have assumed, for simplicity, that parasite disappearance affects all ages evenly. However, since various reports indicate that late-stage parasites are more likely to be targeted for parasite clearance than early parasite stages^14^,^17^, we now assume that parasites older than some age, *x_c_*, are cleared at a rate *c* and that parasites younger than this age are not targeted for clearance. This alters the differential equation (4), to become,
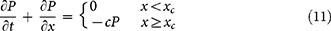
in both our disappearance and parasite growth models. The boundary conditions and the initial conditions of both models are unchanged. The four models are summarised in Supplementary Table 1. The solution of the generalised model when *t* ≤ 1 and with *x_r_* = 1 is presented in Supplementary Information 1.2.

#### Model fitting

All model fitting was performed in MATLAB 7.12.0.635 (R2011a). Fitting was done using the built-in constrained optimising function, “fmincon.m”. Fitting of the disappearance data was performed with a sum-of-squares objective function. In contrast, because of the exponential nature of the parasite growth data, fitting of the growth data was performed using a sum-of-squares objective function on the natural logarithm of all data points and model outputs (Supplementary Information 1.3).

#### Statistical tests

All P-values were evaluated using the non-parametric Mann-Whitney test (“ranksum.m” function in MATLAB 7.12.0.635 (R2011a)).

## Supplementary Material

Supplementary Information Supplementary Information
