# Supplementary material for: Reduced erythrocyte susceptibility and increased host clearance of young parasites slows Plasmodium growth in a murine model of severe malaria
Source: Sci Rep. 2015 May 6;5:9412. doi: 10.1038/srep09412 (PMC5386191; doi:10.1038/srep09412)
Supplement: Supplementary Information [file srep09412-s1.pdf]

Reduced erythrocyte susceptibility and increased host clearance of young parasites slows Plasmodium growth in a murine model of severe malaria

Authors: David S. Khoury, Deborah Cromer, Shannon E. Best, Kylie R. James, Ismail Sebina, Ashraful Haque, Miles P. Davenport*.

Supplementary Information 1: Details of Mathematical Model and Fitting

Supplementary Information 1.1: Viability of donor parasites.

Fluorescently labelled red blood cells (RBCs) containing *Pb*A-GFP$^+$ parasites from donor mice were detectable in recipient mice after transfusion (and a spread of parasite life-stages was detected) (Fig. 2 & 3). However, the ability of the donor parasites to infect the endogenous (recipient) RBCs (i.e. the viability of donor parasites) after transfusion varied depending on the life-stage of the donor parasite (Supplementary Fig. 1). In both the naïve and acutely infected groups of mice, the donor trophozoites and rings had a much larger fold increase over one cycle of reproduction (1 day), compared with the transfused schizonts. Though at first one might hypothesise that the lower fold increase of schizonts could be a result of schizont sequestration, this is not supported by the data. We see that in the 10 hours after donor parasites are transfused into naïve mice, there was very little infection of endogenous RBCs by donor parasites at all (Fig. 2c). Hence, although donor parasites at the schizont stage were transferred into recipient mice (Fig. 3), the donor schizonts produced less progeny after one-cycle of reproduction compared with donor rings and trophozoites. This result suggests that the donor schizonts were less viable (less able to infect other RBCs after rupture) compared with donor rings and trophozoites. One plausible reason for the reduced viability of the late-stage donor parasites is that these mature stages are more fragile and so more susceptible to damage during the labelling and transfusion processes. Hence, labelling and transfusion may

somehow prevent late-stage donor parasites from properly releasing viable merozoites and so hinder their multiplication.

Modelling parasite stage dependent viability. We have taken the reduced viability of late-stage donor parasites after labelling and transfusion into account when modelling the growth of the donor parasite in the recipient host (over a one day reproductive cycle). In our model the action of donor parasites infecting other RBCs upon rupture is described by the boundary condition,

$$P_R(t,0) = \beta(t) P_R(t, x_r). \qquad \text{(S1)}$$

Where $\beta(t)$ represents the average number of new RBCs becoming infected by a rupturing mature parasite at time *t*. x_r is the age of parasite rupture (in all the modelling in this paper x_r is taken to be 1 day). The function $\beta(t)$.is dependent on time due to the varied viability of donor parasites described above. Donor parasites that were transfused into the recipient mice at age, x will rupture at time $t = x_r - x$. For example, a late-stage parasite of age 18 hours, or 0.75 days, will rupture at time $t = 0.25$ days after transfusion. In contrast, an early stage parasite, transfused at an age of 8 hours, or 0.33 days, will rupture at time $t = 0.67$ days after transfusion. By our observation that late-stage donor parasites are less viable than young donor parasites we expect the late-stage parasites to produce fewer new infected RBCs than the early stage parasites, and thus should have a lower associated multiplication rate, β. Allowing $\beta(t)$ to vary with time over the first day of infection will account for the different viability of the parasites.

To estimate $\beta(t)$ we consider infection of endogenous RBCs in the naive recipient mice (Supplementary Fig. 2a) and label these infected recipient cells as *R*(*t*). We observe a relatively simple function can be used to fit the infection of recipient RBCs with *Pb*A-GFP$^+$ parasites,

$$R(t) = Ct^A. \qquad \text{(S2)}$$

The 10-minute time point in Supplementary Fig. 2a has been excluded from the fit, as there should be almost no recipient RBCs infected with donor parasites immediately after transfusion of the donor parasites into the recipient mice. The cells we detect at this time point are consistent with the presence of a small (>0.05%) fraction of RBCs from the donor mice that were unsuccessfully labelled but also contained GFP-parasites. These infected (GFP^+) but unlabelled donor cells, when transfused into the recipient mice were estimated to make up approximately 0.0002% of total RBCs. The median percentage of unlabelled cells with donor parasites at 10 minutes after transfusion is 0.0003% (Supplementary Fig. 2a). These unlabelled donor cells make up a very small fraction of the total RBCs such that by 2 hours after transfusion they seem inconsequential.

We can assume minimal clearance of parasites in naïve mice (since clearance mechanisms have not yet begun), and thus the number of parasites rupturing at time t, $P_R(t, x_r)$, will be $P_R(0, x_r - t)$. That is, the number of parasites there were of age $x = x_r - t$, immediately after donor parasites were transfused into the recipient mice at $t = 0$. Since an approximately uniform distribution of parasite ages was transfused into the recipient mice $P_R(0, x_r - t) = \frac{P_0}{x_r}$, where P_0 the starting number of parasites. Thus we have

$$P_R(t, x_r) = P_R(0, x_r - t) = \frac{P_0}{x_r}. \tag{S3}$$

The number of RBCs becoming infected from a rupturing parasites at time t is $\beta(t)$, and therefore number of recipient cells becoming infected at a time t is given by,

$$R(t) = \int_0^t \frac{P_0}{x_r} \beta(\lambda) d\lambda. \tag{S4}$$

Combining equation (S2) with equation (S4), and solving, we arrive at the estimate for $\beta(t)$,

$$\beta(t) = Kt^{A-1} \tag{S5}$$

where K is a combination of parameters, P_0, A, and C. By fitting the data in Supplementary Fig. 2 we can estimate A~2.6 and C~-1.1. From equation (S5), we observe that the relative viability of parasites by their age at the time of transfusion is given by,

$$v(x) = (x_r - x)^{A-1}, \quad \text{(S6)}$$

which is plotted in Supplementary Fig. 2b. The parasite multiplication rate, β_0, (fold increase of parasite numbers over 1 day) is given by

$$\beta_0 = \int_0^{x_r} K t^{A-1}\, dt = \frac{K}{A} x_r^A \quad \text{(S7)}$$

Combining equations (S5) and (S7) gives us an expression for $\beta(t)$ involving only A, which was estimated from fitting the data in Supplementary Fig. 2a, x_r, the age of parasite rupture and β_0, the parasite multiplication rate,

$$\beta(t) = \beta_0 A \frac{t^{A-1}}{x_r^A}. \quad \text{(S8)}$$

Supplementary Information 1.2: Solution of parasite growth model with non-uniform parasite clearance

The model of parasite growth with a non-uniform clearance rate presented in the main report is presented below, with $x_r = 1$.

$$\frac{\partial P_R}{\partial t} + \frac{\partial P_R}{\partial x} = \begin{cases} 0 & x < x_c \\ -cP_R & x \geq x_c \end{cases} \qquad \text{(S9)}$$

$$P_R(0, x) = P_0 \qquad \text{(S10)}$$

$$P_R(t, 0) = \beta_0\, A\, t^{A-1} P_R(t, 1) \qquad \text{(S11)}$$

Solving this partial differential equation for $t \leq 1$ day using the method of characteristics provides the solution,

$$P_R(t,x) = \begin{cases} P_0 & x \geq t \,\&\, x < x_c \\ P_0 e^{-c(x-x_c)} & x \geq t \,\&\, x \geq x_c \,\&\, x - t \leq x_c \\ P_0 e^{-ct} & x \geq t \,\&\, x - t > x_c \\ \beta_0 A P_0 (t-x)^{A-1} e^{-c(t-x)} & x < t \,\&\, x < x_c \,\&\, t - x < 1 - x_c \\ \beta_0 A P_0 (t-x)^{A-1} e^{-c(1-x_c)} & x < t \,\&\, x < x_c \,\&\, t - x \geq 1 - x_c \\ \beta_0 A P_0 (t-x)^{A-1} e^{-c(t-x_c)} & x < t \,\&\, x \geq x_c \end{cases}$$

Supplementary Information 1.3: Least squares fitting

Fitting was performed using the built-in constrained optimising function "fmincon.m", available with the optimization toolbox in MATLAB 7.12.0.635 (R2011a). This function minimises the output of a user-provided function. In the case of our model fitting, we constructed a sum-of-squares objective function for fitting the parasite clearance data with the clearance model, and a sum-of-squares of the natural log of the data and model outputs when fitting the parasite growth model.

The models were fit to each mouse individually to estimate parameters P_0 and x_c. In models that included the other parameters β_0 and x_r, the model was fit to the data with a series of fixed values for these two parameters, and choice of β_0 and x_r which produced the best fit for all mice (that is, the sum-of-squares for each mouse added together) were used to understand the data.

Supplementary Figures and Tables

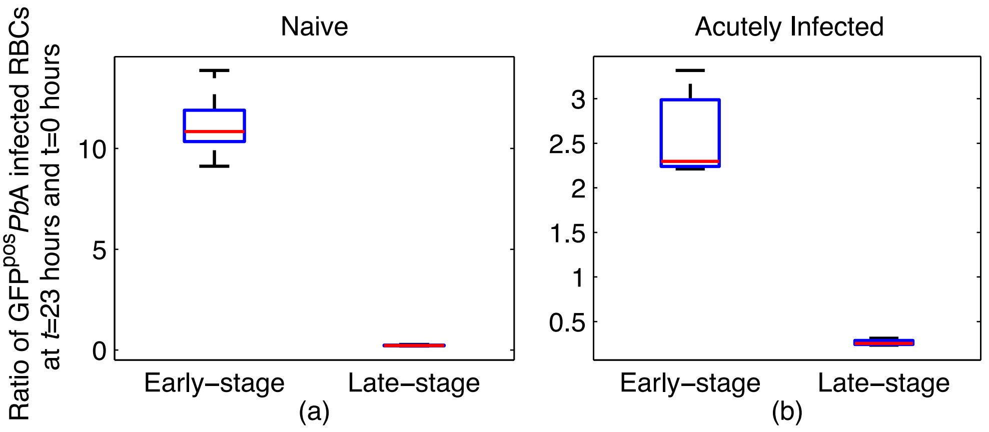

Supplementary Figure 1: Comparing growth of donor parasites at different ages. We compared the proportion of total red blood cells that were infected with young (rings or trophozoites) and mature (schizonts) donor parasites immediately after transfusion, with the proportions 23 hours later. We observed that the young parasites that were transfused into the recipient mice multiplied much more than the mature stage parasites that were transfused into mice, in both naïve (a) and acutely infected mice (b). The number of mature stage parasites decreases (ratio < 1) in both groups of mice, however the number of early-stage parasites increases by 10.8 fold (median) in naïve mice (a), and 2.3 fold in acutely infected mice (b) over the 23-hour time period.

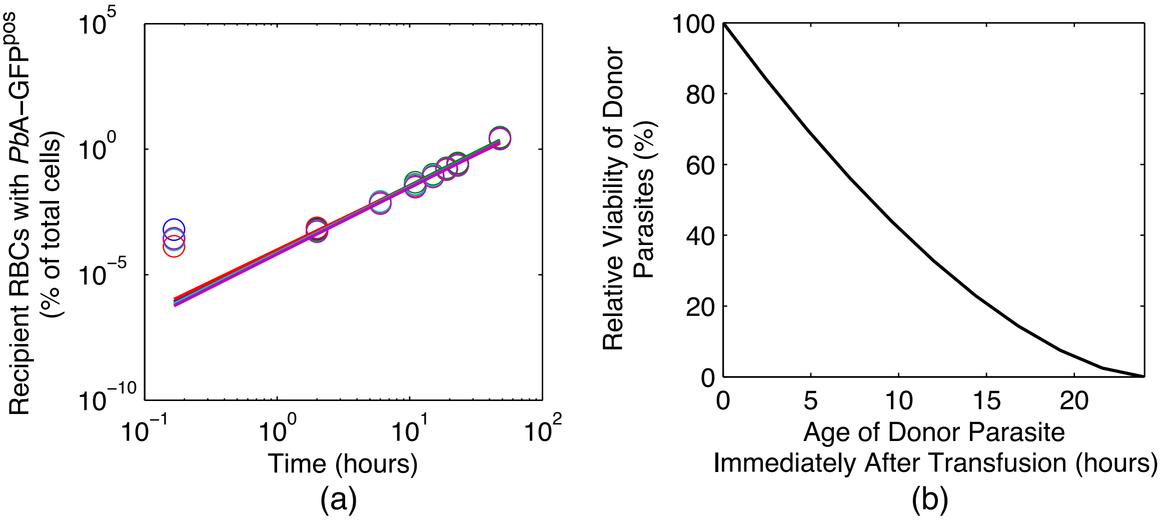

Supplementary Figure S2: Parasite viability decreases with the age of the parasite at the time of transfusion. As the donor parasites rupture within the recipient hosts the donor parasite infects

recipient RBCs in the recipient mice. The circles in (a) show the percentage of total cells that are recipient cells infected with donor parasites in naïve mice. The solid lines show the fits obtained when fitting equation (S10) to this data. The first time point (10 minutes post transfusion) is excluded from the fits as there should be almost no recipient RBCs infected with donor parasites directly after the donor parasites are introduced to the recipient mice, these cells are predominantly transfused cells infected with GFP-parasites that were unsuccessfully labelled. The data and these fits indicate how the rate of infection of RBCs is increasing over time. We use these fits to estimate how the rate of infection changes with the age of parasites at the time of transfusion. We find that the variation in the viability of parasites with age is given by the equation $v(x) = (x_r - x)^{A-1}$, where x is age, $x_r = 1$, and $A = 2.6$ and is shown in (b).

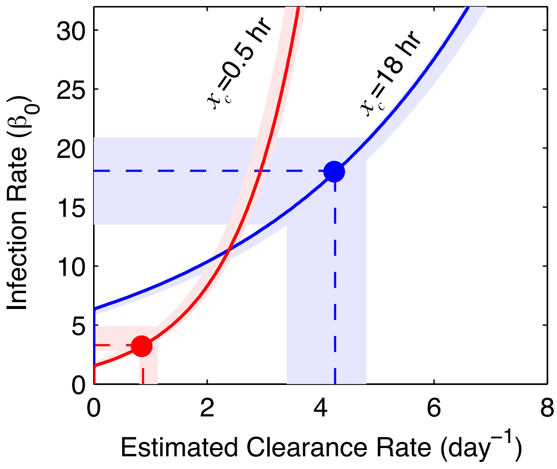

Supplementary Figure S3: Estimating the rate of infection in naïve and acutely infected mice. In figure 3c in the main text we showed that, when assuming uniform rate of parasite clearance in mice and after taking into account the higher parasite clearance rate in acutely infected mice compared to naïve mice, the rate of infection of red blood cells is lower in acutely infected mice. Here we show that even after considering the stage-specific nature of parasite clearance, we again estimate a reduced rate of infection of red blood cells in acutely infected mice compared with naïve mice. The age of parasite clearance chosen for each group in this figure was estimated by fitting the model of age-dependent parasite clearance (section 1.1) to the data on parasite disappearance (Fig. 2d). The clearance age used for each group of mice were $x_c = 18$ and $x_c = 0.5$ hrs for naïve and

acutely infected mice respectively. When estimating the age of parasite clearance for each group, we also obtained an estimate of the parasite clearance rate for each group of mice (the medians are shown as the dashed lines for each group). The solid lines indicate the clearance rate of parasites that is estimated when the parasite growth data is fitted with a model of parasite growth, with a fixed rate of infection of red blood cells (β_0), and a fixed age of parasite clearance (as estimated for each group). The points of intersection of the solid and dashed lines for each group of mice provide an estimate of the parasite multiplication rate β_0 for each group of mice. Hence, we observe that a lower parasite multiplication rate is estimated in the acutely infected mice than in the naïve mice.

Supplementary Table 1: Summary of Mathematical Models

Model for fitting data on:	*Disappearance of donor cells infected with donor parasites*	*Disappearance of donor cells infected with donor parasites*	*Growth of donor parasites in donor and endogenous RBCs*	*Growth of donor parasites in donor and endogenous RBCs*
Type of clearance used in model:	*Uniform clearance*	*Age-dependent clearance*	*Uniform clearance*	*Age-dependent clearance*
Partial Differential equation	$\frac{\partial P}{\partial t} + \frac{\partial P}{\partial x} = -c(x)P$			
Disappearance function, $c(x)$	*Constant,* c	$\begin{cases} 0 & x < x_c \\ c & x \geq x_c \end{cases}$	*Constant,* c	$\begin{cases} 0 & x < x_c \\ c & x \geq x_c \end{cases}$
Initial Condition, $P(0,x)$	*Constant,* P_0	*Constant,* P_0	*Constant,* P_0	*Constant,* P_0
Boundary Condition, $P(t,0)$	*0*	*0*	$\beta(t)P(t,x_r)$	$\beta(t)P(t,x_r)$
